# Indocyanine green is a sensitive adjunct in the identification and surgical management of local and metastatic hepatoblastoma

**DOI:** 10.1002/cam4.3982

**Published:** 2021-06-12

**Authors:** Charissa M. Lake, Alexander J. Bondoc, Roshni Dasgupta, Todd M. Jenkins, Alexander J. Towbin, Ethan A. Smith, Maria H. Alonso, James I. Geller, Gregory M. Tiao

**Affiliations:** ^1^ Division of Pediatric General and Thoracic Surgery Cincinnati Children’s Hospital Medical Center Cincinnati OH USA; ^2^ Department of Radiology Cincinnati Children’s Hospital Medical Center University of Cincinnati College of Medicine Cincinnati OH USA; ^3^ Division of Pediatric Oncology Cincinnati Children’s Hospital Medical Center Cincinnati OH USA

**Keywords:** hepatectomy, hepatoblastoma, indocyanine green, metastasectomy, pediatric surgery, thoracoscopy, thoracotomy

## Abstract

**Background:**

Hepatoblastoma is the most common primary pediatric liver malignancy. Indocyanine green (ICG) has been described as an adjunct to resection in small series. Its utility remains undefined in larger cohorts.

**Methods:**

Records for 29 patients diagnosed with hepatoblastoma who received ICG prior to surgical resection from 2017 to 2020 at a single institution were retrospectively reviewed. The primary outcome was correlation between intraoperative ICG‐avidity and histologic presence of hepatoblastoma. A secondary outcome included the histologic margin designation for resected liver specimens.

**Results:**

ICG sensitivity was 91% for 120 resected thoracic specimens from 21 patients. Specificity was 57%. In 10% of operations, HB‐positive specimens were resected solely on ICG‐avidity. In an additional 40% of cases, ICG assisted in localizing a preoperatively diagnosed lesion. ICG sensitivity during thoracotomy and thoracoscopic surgery was 95 and 74%, respectively; primary and relapsed disease demonstrated sensitivity of 94 and 73%, respectively. Sensitivity was 92% for 25 resected liver specimens from nine patients with all parenchymal margins grossly negative for disease. Four multifocal lesions were identified with two resected solely by ICG‐avidity.

**Conclusions:**

ICG is a sensitive adjunct for identifying local and metastatic hepatoblastoma, including lesions not visualized on preoperative imaging, and delineating margins during liver resection. False positives limit specificity; however, there were no adverse outcomes from additional resections. We noted that thoracoscopic surgery can be completed safely in patients with less significant disease burden, and conversion to thoracotomy, if necessary, is straightforward.

## Graphical abstract

ICG is a sensitive adjunct for identifying local and metastatic hepatoblastoma, including lesions not visualized on preoperative imaging, and delineating margins during liver resection. With its use, thoracoscopic surgery can be completed safely in patients with less significant disease burden with conversion to thoracotomy, if necessary.

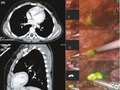

**Lay Summary**

Identifying the margins of a cancer for removal or distant disease that has spread can be difficult and fluorescent dye has been explored as a way to improve identification of disease. Cancer cells take up the dye allowing them to be identified, but the degree is dependent on the type of cancer. In hepatoblastoma, the most common pediatric liver cancer, the fluorescent dye indocyanine green (ICG) can assist in the identification of liver disease and distant disease, including lesions not seen on preoperative imaging. This approach may improve the removal of all cancer at the time of operation.

## 1 INTRODUCTION

Hepatoblastoma (HB) is the most common primary pediatric hepatic malignancy with an increasing incidence worldwide.^1^, ^2^ Improvements in surgical approach and chemotherapy have increased overall survival (OS) to over 70%^1^, ^3^, ^4^, ^5^ ; however, 10%–20% of patients with metastatic HB experience inferior outcomes.^6^, ^7^, ^8^, ^9^, ^10^ Preoperative axial imaging identifies lesions, margins, and vascular or local invasion. Surgical options for local control include conventional resection and/or liver transplantation. Pulmonary metastatic disease is initially treated with chemotherapy, and if persistent, subsequent thoracic exploration. Visual inspection, palpation, and radiographic‐guided needle localization identify metastatic lung lesions.

Indocyanine green (ICG) is a fluorescent anion excited at a wavelength of 750–810 nm by the near infrared spectrum and fluoresces at 830–840 nm.^11^, ^12^, ^13^, ^14^ ICG has been utilized during surgical procedures to identify malignancies.^11^, ^12^ Intraoperatively, imaging systems detect ICG fluorescence in tissue using open or minimally invasive approaches.^15^ In the case of HB, ICG has been used to detect tumor cells that retain ICG due to its pharmacodynamic properties. Specifically, hepatic uptake occurs with eventual excretion into bile. However, due to morphologic changes of HB cells, ICG excretion is decreased and can facilitate detection of both primary and metastatic disease.^15^

Our institution began using ICG in 2017 at the time of both hepatectomy and pulmonary metastasectomy with the hypothesis that ICG would (1) augment lesion identification and (2) ensure adequate resection with negative margin. In this study, we report ICG’s adjunctive efficacy in the surgical management of both metastatic and primary hepatoblastoma.

## 2 METHODS

A retrospective review, approved by the Institutional Review Board (IRB) at the author's institution (#2018‐3321, #2020‐0386) and in accord with the ethical standards of the Helsinki Declaration of 1975, authorized collection of preoperative, peri‐operative, and postoperative data from patients with HB who received ICG prior to operative management between June 2017 and April 2020 from the institution's electronic health record.

For metastatic disease, the number of thoracic lesions was determined independently by two certificate of added qualification‐certified pediatric radiologists blinded to clinical parameters. Miliary disease was defined as greater than 10 nodules in a unilateral lung. Pathologic assessment of operative specimens documented presence of malignancy.

Patients undergoing liver resection received ICG intravenously at a dose of 0.5 mg/kg administered 1–6 days prior to surgery. Patients undergoing metastasectomy received ICG intravenously at a dose of 0.5 mg/kg 24 h prior to surgery. ICG was dispensed and administered directly by the attending surgeon of record. The type of approach (‐open vs minimally invasive‐) was at the discretion of the operating surgeon with all procedures performed by three surgeons. Due to equipment availability, the first seven patients with thoracic disease underwent eight procedures utilizing thoracotomy. Patients with bilateral disease underwent staged procedures.

The ICG detection camera utilized for the first eight cases was the Hamamatsu pde‐neo (Mitaka USA). The Stryker SPY‐PHI and 1588‐AIM system (Stryker Corporation) were utilized for all subsequent operations. Lesions were characterized ICG‐positive or ICG‐negative during surgery both in situ and ex vivo after resection. ICG‐avidity was not quantified as the technology for objective quantification does not exist.

### 2.1 Statistical analysis

Advanced statistics were performed with SAS v9.4. Generalized linear mixed modeling evaluated the relationship between ICG positivity and pathologically proven HB, accounting for multiple within‐patient surgeries and histologies. Marginal Cox proportional hazards modeling assessed ICG presence with OS and event‐free survival (EFS), accounting for multiple within‐patient surgeries. Hazard ratios (HR) and 95% confidence intervals (CI) were generated from these models. Linear mixed modeling assessed the relationship between ICG presence with log‐transformed number of days hospitalized (HD) accounted for multiple within‐patient surgeries; log‐transformed number of days on surgical service (SS), number of days with a chest tube (ChT), and age at surgery were also generated. Geometric means and 95% CI were obtained from this model. The other descriptive statistics and Kaplan–Meier Survival curves were generated from GraphPad 8.4.1

## 3 RESULTS

### 3.1 Thoracic disease

Twenty‐one patients underwent 29 operations for metastasectomy. Demographic data is summarized in Table 1. Twelve operations were completed thoracoscopically, nine were converted from thoracoscopy to thoracotomy, and eight performed via thoracotomy. The most common reason for conversion was inability to visualize or palpate lesions thoracoscopically. Multiple lesions sometimes necessitated conversion to thoracotomy.

**TABLE 1:** Patient and disease characteristics, monitoring, treatment type, and outcome for thoracic operations

Patient Number	Age (years)/Gender	Preoperative Chemotherapy (of cycles)	Type of Surgery	Primary (P) v. Relapse (R)	Number Specimens Resected/ ICG+/HB+	Number of Chest Tube Days	Number of Hospital Days			Complications	AFP at Surgery/30 days Postop	Follow‐up Days	Outcome
							ICU	Surg	Onc				
**1**	3.64/F	Cisplatin/vincristine/5‐FU x6 Carboplatin/doxorubicin x2 Ifosfamide/etoposide x2 Vincristine/irinotecan x6 Cisplatin x2 Gemcitabine/docetaxel x1	Thoracotomy	R	1/1/1	2	4				7.3/2.1	886	Alive without disease
							0	4	0				
**2**	2.8/M	Cisplatin/doxorubicin x3 Carboplatin/doxorubicin x3 Vincristine/irinotecan x3 Ifosfamide/carboplatin/etoposide x1	Thoracotomy	R	4/4/1	2	10				776/21.4	304	Deceased
							0	10	0				
**3**	2/F	Vincristine/irinotecan x3 Cisplatin/vincristine/doxorubicin/5‐FU x5 Ifosfamide/carboplastin/etoposide x4 Pazopanib Gemcitabine/docetaxel x3 Cisplatin x2	Thoracotomy	R	1/1/1	1	4				46/13.4	792	Alive without disease
							0	4	0				
**4**	6.59/M	Cisplatin/doxorubicin x3 Carboplatin/doxorubicin x3 Cisplatin/vincristine/5‐FU x2 Ifosfamide/carboplatin/etoposide x3 Irinotecan x6	Thoracotomy	R	3/0/1	2	5				1.8/1.7	756	Alive without disease
							1	4	0				
**5**	4.58/M	Cisplatin x1 Carboplatin x1 Cisplatin/doxorubicin x7 Sorafenib	Thoracotomy	P	8/8/8	2	242			Transfusion	7345.5/2971.1	47	Deceased
							0	0	41				
**6**	6.5/M	Cisplatin/vincristine/5‐FU x2	Thoracoscopy to Thoracotomy	R	1/0/0	1	3				1.7/^a^	434	Alive without disease
							0	3	0				
**7a**	6.91/M	Cisplatin/doxorubicin x3	Thoracotomy	P	7/0/0	2	5				15928./104814	171	Deceased
							0	5	0				
**7b**	6.93/M	^b^	Thoracotomy	P	2/0/0	2	5				^c^/^c^		
							0	5	0				
**8**	6.5/M	Cisplatin/vincristine/5‐FU x8 Carboplatin/doxorubicin x2	Thoracoscopy	R	2/2/2	0	3				335.1/26.7	512	Alive with disease
							0	3	0				
**9a**	3.59/F	Cisplatin/doxorubicin x2	Thoracoscopy to Thoracotomy	P	6/6/5	2	66				2499.1/1669	371	Alive without disease
							0	2	15				
**9b**	3.66/F	Cisplatin/doxorubicin x1	Thoracoscopy	P	3/3/1	1	25				1695.8/1115.6		
							0	1	16				
**9c**	3.76/F	Carboplatin/doxorubicin x1	Thoracoscopy	P	3/2/3	5	38				850.6/27.5^d^		
							2	4	3				
**10a**	2.96/M	Vincristine/irinotecan/tersirolimus x2 Cisplatin/vincristine/doxorubicin/5‐FUx6 Carboplatin/etoposide x1 Cisplatin x1	Thoracoscopy to Thoracotomy	R	2/2/2	1	4			Transfusion	430/233.4	48	Alive without disease
							1	3	0				
**10b**	3.08/M	^b^	Thoracoscopy	R	2/0/2	1	3				^c^/^c^		
							0	3	0				
**11a**	1.85/M	Cisplatin/doxorubicin x3 Carboplatin/doxorubicin x3 Carboplatin/etoposide x1	Thoracoscopy	P	1/1/0	1	33				1321.8/1608.3	377	Alive without disease
							0	6	10				
**11b**	1.86/M	^b^	Thoracoscopy	P	5/2/3	1	same admission as 11a				^c^/^c^		
		
**12**	1.47/F	Cisplatin x5	Thoracoscopy to Thoracotomy	P	1/0/0	1	744			Tracheitis Transfusion	275.8/14.7^d^	314	Alive without disease
							2	0	9				
**13a**	1.83/F	Cisplatin/doxorubicin x3 Carboplatin/doxorubicin x1	Thoracoscopy	P	4/4/3	1	3				12.8/2.7	137	Alive without disease
							0	3	0				
**13b**	1.93/F	Carboplatin/doxorubicin x1	Thoracoscopy	P	1/0/0	1	3				2.7/10.7		
							0	3	0				
**14**	1.7/F	Vincristine/irinotecan/tersirolimus x2 Cisplatin/doxorubicin x2 Carboplatin/doxorubicin x1	Thoracoscopy to Thoracotomy	P	4/1/1	3	6				4.3/5	56	Alive without disease
							2	3	0				
**15a**	1.88/M	Vincristine/irinotecan x3 Cisplatin/vincristine/doxorubicin/5‐FUx6	Thoracoscopy to Thoracotomy	P	7/6/7	2	4				1573.1/2953.1	230	Deceased
							1	3	0				
**15b**	2.06/M	Ifosfamide/carboplatin/etoposide x2	Thoracoscopy to Thoracotomy	P	5/5/5	1	8				2953.1/2692.4		
							0	2	6				
**16**	3.24/M	Cisplatin/vincristine/doxorubicin/5‐FUx6	Thoracoscopy	R	1/1/1	1	4				5624.7/4538.3	197	Alive with disease
							0	4	0				
**17**	2.03/F	Cisplatin/doxorubicin x3 Carboplatin/doxorubicin x3	Thoracoscopy	P	2/0/0	1	2				8/^a^	3	Alive without disease
							0	2	0				
**18a**	2.34/M	Cisplatin/doxorubicin x3 Vincristine/irinotecanx2	Thoracoscopy to Thoracotomy	P	20/20/19	2	4				153.7/75.3	133	Alive with disease
							0	4	0				
**18b**	2.59/M	Cisplatin/doxorubicin x1	Thoracotomy	P	17/17/15	2	4				15.9/11.9		
							2	2	0				
**19**	3.65/M	Cisplatin/vincristine/doxorubicin/5‐FUx6 Vincristine/irinotecan x7 Ifosfamide/carboplatin/etoposide x2	Thoracoscopy to Thoracotomy	P	3/3/0	1	7				9.9/444	80	Alive with disease
							0	4	0				
**20**	11.54/M	Cisplatin/doxorubicin x3 Carboplatin/doxorubicin x3 Vincristine/irinotecan x4 Ifosfamide/etoposide x8	Thoracoscopy	R	1/1/1	1	3				226.3/7.8	74	Alive without disease
							0	3	0				
**21**	2.26/F	Cisplatin/doxorubicin x3 Carboplatin/doxorubicin x3 Carboplatin/etoposide x1	Thoracoscopy	R	3/2/3	1	3			Pneumothorax requiring chest tube placement	187.6/34	66	Alive without disease
							0	3	0				

If the patient underwent multiple operations, the operations are listed in chronological order designated by a letter (a, b, c) following the patient number. The preoperative chemotherapy regimens indicate the total number of cycles received of each regimen, but are not in chronological order. Serum alpha‐fetoprotein (AFP) levels (ng/ml) preoperatively and 30 days postoperatively are provided where available with notations made if an additional oncologic surgery was performed prior to the post‐operative result. Number of hospital days indicates the total number of days the patient was admitted for the hospitalization wherein the surgery occurred. Days on the ICU, surgery and oncology services indicate postoperative days on these services, exclusively. Under complications, transfusion indicates that the patient received a blood transfusion within 72 hours following his/her operation. Follow‐up duration is calculated from the day of surgery or the day of first surgery for patients who underwent multiple procedures.

^a^ 30 day postoperative AFP not available.

^b^ No additional chemotherapy between operations.

^c^ Operation has the same preoperative and 30‐day and/or postoperative AFP as the surgery above.

^d^ Patient had a transplant prior to the 30‐day postoperative AFP.

#### 3.1.1 Preoperative imaging and operative resection

Computed Tomography (CT) imaging was obtained 2 weeks prior to surgery (mean of 13.34 days). Median number of lesions on preoperative imaging was three (range, 0–59). Lesions were reported by lung laterality and lobe (Table 2). Mean number of specimens resected per operation was 4.14 (range, 1–20). On pathological examination, most contained one lesion per specimen, but the maximum recorded was 28 lesions in a single lobectomy specimen.

**TABLE 2:** Total and lobar distribution of nodules on preoperative imaging, intraoperatively, and by pathology for thoracic lesions

Patient Number	Pre‐operative Imaging			Intra‐operative						Pathology					
	Total Number of Lesions: Imaging			Total Number of Specimens Resected			Total Number of Nodules Resected			Total Number of Lesions on Pathology			Total Number with HB		
	RUL	RML	RLL	RUL	RML	RLL	RUL	RML	RLL	RUL	RML	RLL	RUL	RML	RLL
1	**3**			**1**			**1**			**1**			**1**		
	1	1	1	1	0	0	1			1			1		
3	**4**			**1**			**1**			**1**			**1**		
	1	1	2	1	0	0	1			1			1		
4	**1**			**3**			**0**			**2**			**2**		
	1	0	0	3	0	0				2			2		
5	**26**			**8**			**10**			**8**			**8**		
	5	9	12	1	4	3	1	7	2	1	4	3	1	4	3
6	**3**			**1**			**1**			**1**			**0**		
	3	0	0	1	0	0	1			1					
7b	**0**			**2**			**2**			**0**			**0**		
				1	1	0	1	1							
9a	**10**			**6**			**8**			**8**			**8**		
	3	7	0	1	0	5	1		7	2		6	2		6
9c	**1**			**3**			**3**			**4**			**4**		
	1	0	0	1^a^	0	2	1		2	1		3	1		3
10a	**2**			**2**			**2**			**2**			**2**		
	1	1	0^b^	2	0	0	2			2			2		
11a	**1**			**1**			**1**			**0**			**0**		
	1	0	0	0	1	0		1							
12	**3**			**1**			**0**			**0**			**0**		
	0	1	2	0	0	1									
13a	**7**			**4**			**4**			**3**			**3**		
	4	2	1	1	1	2	1	1	2	1	1	1	1	1	1
14	**4**			**4**			**2**			**1**			**1**		
	0^c^	0	4	1	1	2	0	0	2	1			1		
15a	**10**			**7**			**13**			**10**			**10**		
	5	1	4	1	2	4	4	3	6	3	2	5	3	2	5
16	**1**			**1**			**1**			**1**			**1**		
	1	0	0	0	1	0		1			1			1	
17	**3**			**2**			**2**			**0**			**0**		
	2	0	1	1	1	0	1	1							
18b	**59**			**17**			**16** ^d^			**44**			**44**		
	5	14	40	6	10	1	6	10	TNTC^d^	6	10	28	6	10	28
19	**12**			**3**			**3**			**3**			**0**		
	6	3	3	3	0	0	3			3					
															

Patient Number	Pre‐operative Imaging			Intra‐operative						Pathology					
	Total Number of Lesions: Imaging			Total Number of Specimens Resected			Total Number of Nodules Resected			Total Number of Lesions: Pathology			Total Number with HB		
	LUL	LLL		LUL	LLL	Other	LUL	LLL	Other	LUL	LLL	Other	LUL	LLL	Other
2	**2**			**4**			**4**			**4**			**1**		
	1	1		2	1	1^e^	2	1	1	1	1	2		1	
7a	**3**			**7**			**6**			**0**			**0**		
	3	0		3	3	1^e^	2	3	1						
8	**0**			**2**			**2**			**3**			**2**		
				0	0	2^f^			2			3			2
9b	**4**			**3**			**3**			**1**			**1**		
	0	4		1	2	0	1	2			1			1	
10b	**3**			**2**			**1**			**2**			**2**		
	0	3		0	2^a^	0		1			2			2	
11b	**0**			**5**			**5**			**3**			**3**		
				3	2	0	3	2		2	1		2	1	
13b	**8**			**1**			**1**			**0**			**0**		
	1	7		0	1^a^	0	0	1							
15b	**10**			**5**			**9**			**10**			**10**		
	3	7		3	2	0	4	5		6	4		6	4	
18a	**40**			**20**			**25** ^d^			**41**			**41**		
	17	23		9	11	0	12^d^	13^d^		18	23		18	23	
20	**1**			**1**			**1**			**1**			**1**		
	1	0		1	0	0	1			1			1		
21	**4**			**3**			**3**			**3**			**3**		
	2	2		2	1	0	2	1		2	1		2	1	

Preoperative images were determined by two blinded radiologists as a summary, they did not evaluate extra‐pleural disease. In the intraoperative setting, a nodule was present if it was directly visualized or palpated, or was ICG‐avid. When the number of intraoperative nodules resected was 0, the specimen was removed based on CT imaging or wire localization without the a visible, palpable or ICG‐avid lesion. The total number of lesions on pathology was determined by gross and microscopic evaluation and noted in the pathology reports, where they also noted if HB malignancy was present.

^a^ wire localization utilized during the procedure.

^b^ the corresponding lung lobe was previously resected and therefore not present on imaging.

^c^ suspected tumor thrombus, but no discrete lesions.

^d^ the number listed is the number of discrete lesions recorded, however in these samples there were additional lesions that were not quantified in the operative notes.

^e^ the lesions were described as lymph nodes in the operative report.

^f^ pericardial and chest wall lesions. TNTC – too numerous to count.

For nine operations in eight patients, more lesions were removed than initially determined on preoperative imaging. Pathologic analysis verified HB in seven of eight patients. Five operations on five patients found the exact number of lesions noted on imaging; however, in most surgeries, the lobar distribution of resected nodules differed from preoperative notation. All but one patient in this group had HB on final pathology. In the remaining 15 procedures in 13 patients, fewer nodules were found intraoperatively than noted on preoperative scans. (Table 2).

#### 3.1.2 Pathologic evaluation

**FIGURE 1:**
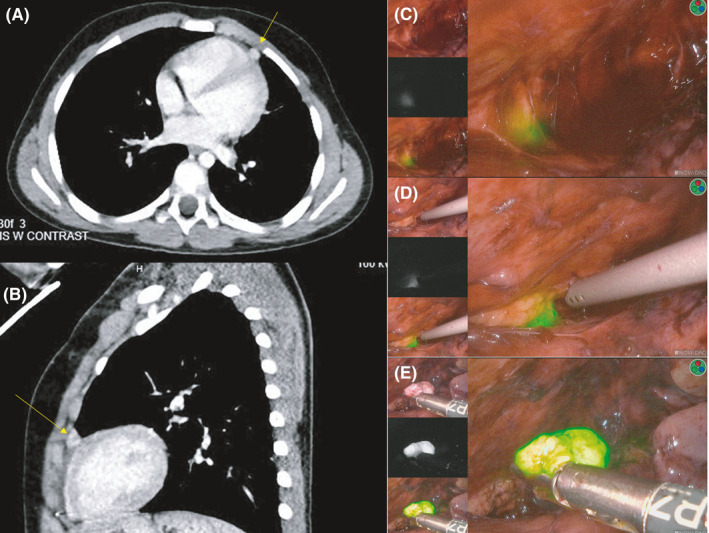
CT and intraoperative imaging of HB pericardial lesion. The left panel (A‐B) demonstrates the preoperative CT in axial (A) and sagittal (B) views. The lesion is anterior to the heart, indicated by the yellow arrow. The right (C‐E) panel shows the intraoperative imaging during thoracoscopy. Each of the intraoperative images shows the three views available in the operating room with the Pinpoint ICG system (top – normal camera view, middle – black and white with ICG avidity appearing white, bottom/right – ICG overlay view where ICG‐avidity appears green). The top image (C) shows the initial visualization of the pericardial lesions and the following images (d&e) demonstrate the increasing ICG avidity with progressive dissection and mobilization. This highlights the penetration depth of ICG and the visual enhancement accomplished with additional dissection

**FIGURE 2:**
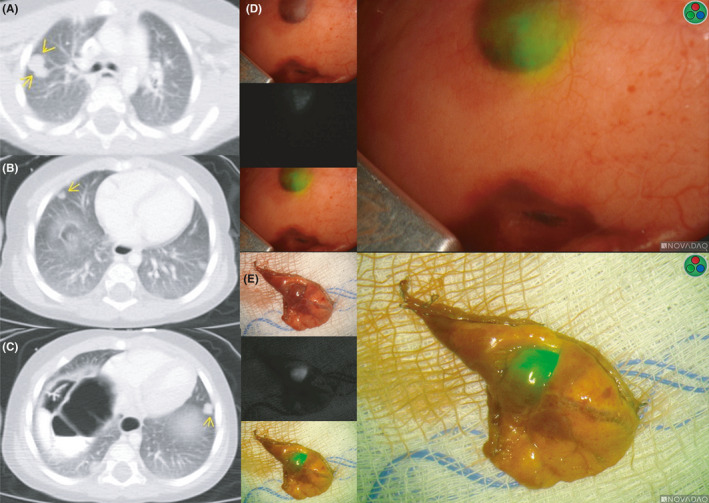
CT and intraoperative imaging of ICG‐positive lung lesions. The left panel (A‐C) shows imaging at three different levels showing multifocal bilateral pulmonary disease indicated by the yellow arrows. The right panel (D‐E) demonstrates ICG‐avid lesions during a thoracotomy in‐situ (D) and ex‐vivo (s). The three intraoperative views are in the same orientation described in Figure 1

**FIGURE 3:**
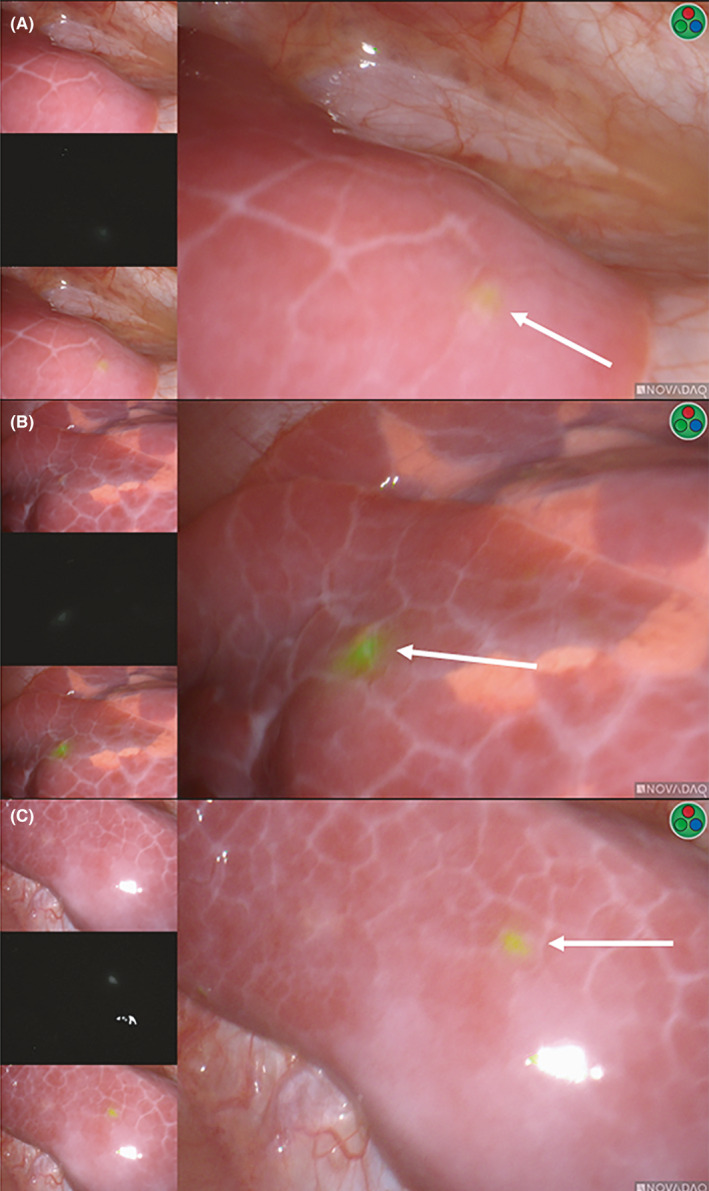
ICG‐avidity of thoracic HB‐positive and HB‐negative lesions. The thoracic ICG‐avid lesions pictured originated from the same patient. Panels A‐B demonstrate true positive (TP) lesions while panel c displays a false positive (FP). The diagnosis associated with the false positive was a giant cell reaction. The white arrows highlight the location of the ICG‐avid lesion

One hundred twenty total specimens were resected, 85 containing hepatoblastoma. Seventy‐seven pathologically positive specimens were ICG‐avid, yielding a sensitivity of 90.6% (Figure 1, Figure 2). Fifteen pathologically negative specimens were ICG‐avid, yielding a specificity of 57.1% (Figure 3). Positive and negative predictive value (PPV and NPV) are 83.7% and 71.4%, respectively. The odds‐ratio (OR) for ICG‐avidity to pathologic positivity was 5.96 (CI 1.40–25.40; p‐value=.02). The most common pathologic diagnosis associated with false positives (FP) included local areas of inflammation (Table 4). The pathologically positive lesions ranged in size from <1 mm to 20 mm; the <1 mm lesion was ICG‐positive. (Table 3).

**TABLE 3:** Diagnostic accuracy of ICG

	Sensitivity	Specificity	Positive Predictive Value	Negative Predictive Value
	% (No.)	% (No.)	% (No.)	% (No.)
Thoracic	91 (77/85)	57 (20/35)	84 (77/92)	71 (20/28)
Thoracotomy	95 (63/66)	58 (15/26)	85 (63/74)	83 (15/18)
Thoracoscopy	74 (14/19)	56 (5/9)	78 (14/18)	50 (5/10)
Primary	94 (66/70)	59 (17/29)	85 (66/78)	81 (17/21)
Relapse	73 (11/15)	50 (3/6)	79 (11/14)	43 (3/7)
PDE‐Neo	92 (11/12)	80 (12/15)	79 (11/14)	92 (12/13)
Stryker	90 (66/73)	40 (8/20)	85 (66/78)	53 (8/15)
SPY‐PHI	96 (52/54)	27 (3/11)	87 (52/60)	60 (3/5)
1588‐AIM	74 (14/19)	56 (5/9)	78 (14/18)	50 (5/10)
Abdominal	92 (12/13)	17 (2/12)	55 (12/22)	67 (2/3)

Sensitivity (SN), specificity (SP), as well as positive and negative predictive value (PPV & NPV) are reported for all thoracic and abdominal patients. Additionally, surgery type, disease subtype and camera system and type are specified for thoracic procedures. The Stryker SPY‐PHI is utilized during thoracotomy while 1588‐AIM is used for thoracoscopy.

**FIGURE 4:**
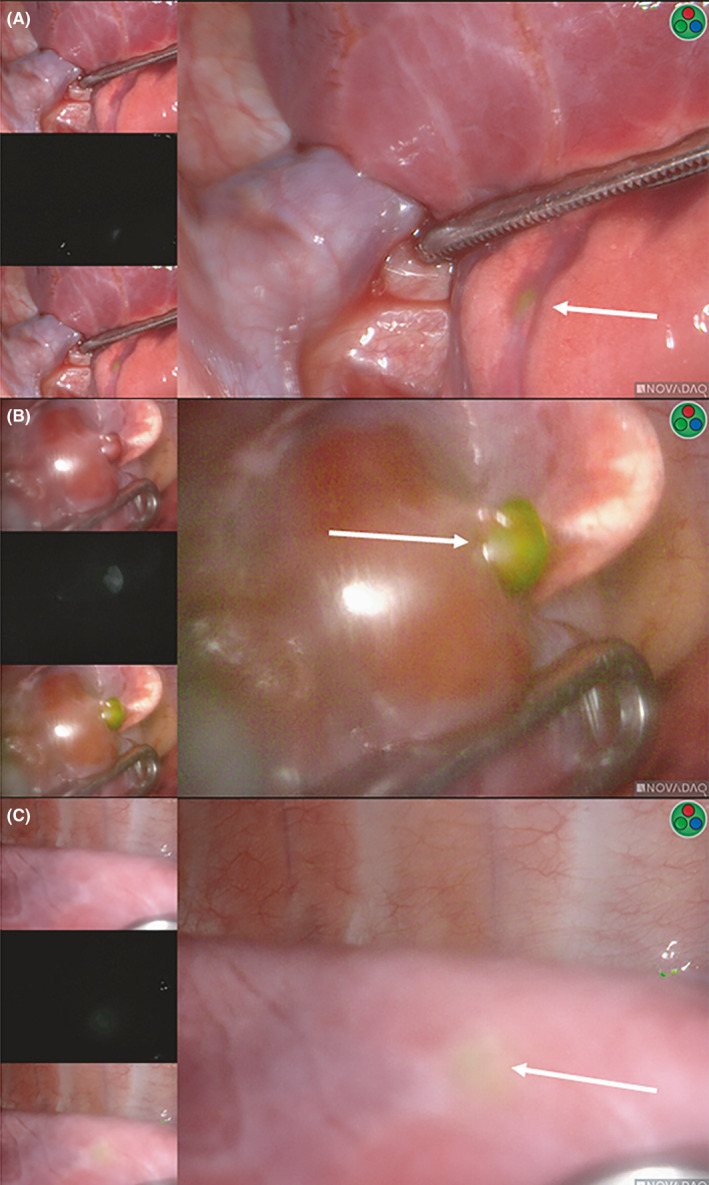
ICG‐avid thoracic lesions not detected on preoperative imaging. Panels A‐C demonstrate thoracic lesions that were ICG‐avid and HB‐positive but not detected on preoperative imaging. Panel B was visible without ICG intraoperatively, while panels A, C were not visible without ICG. The white arrows highlight the location of the ICG‐avid lesion

In 18 procedures, at least one specimen was resected based on ICG‐avidity alone, i.e., lesions neither visible nor palpable. In 14 of 18 procedures, the specimen was pathologically positive. In three patients, lesions were not visualized on preoperative imaging (Figure 4). Of the remaining patients, ICG‐avidity correlated with preoperative imaging.

There were 20 true negative (TN) lesions that were resected during eight procedures on seven patients. Nineteen TN specimens were resected based on correlation with preoperative imaging. Ten of the nodules were palpable, two were palpable and visible on the parenchyma, and one was solely visible. The reason why the final specimen was removed was not specified. The associated diagnoses are reported in Table 4.

**TABLE 4:** Histologic Characteristics of Resected Thoracic Specimens

Patient Number	Total # Specimens	ICG+/HB+ (#)	Subtypes	Viability (%)	ICG‐/HB+ (#)	Subtypes	Viability (%)	ICG+/HB‐ (#)	Diagnoses	ICG‐/HB‐ (#)	Diagnoses
**1**	1	1	Epitheliod, mesenchymal		0			0		0	
**2**	4	1	Fetal, embryonal, pleomorphic		0			3	Reactive lymph nodes, small arterials with thrombi, giant cell reactions, focal chronic bronchiolitis	0	
**3**	1	1	Fetal		0			0		0	
**4**	3	0			1	Predominant Fetal		0		2	Acute/chronic bronchopneumonia; reactive LN
**5**	8	8	Hepatocellular neoplasm ‐NOS		0			0		0	
**6**	1	0			0			0		1	Inflammatory myofibroblastic tumor with foci of new & organizing arterial thrombi
**7**	9	0			0			0		9	Organizing fibrous tissue; intravenous refractile foreign material; type II pneumocytes; focal sinus histiocytosis; organizing pneumonia; focal subpleural atelectasis; subpleural lymphoid aggregate
**8**	2	2	Epithelial	Viable	0			0		0	
**9**	12	8	Trabecular; not specified	30–100	1	Not specified	0	3	Normal lung parenchyma; focal areas of congestion	0	
**10**	4	2	Small cell, embryonal, fetal	90–100	2	Fetal, embryonal; not specified	100	0		0	
**11**	6	2	Mesenchymal	Viable	1	Mesenchymal	Viable	1	Type 2 pigment laden macrophages, small vein with necrotic material	2	Focal hyalinized subpleural fibrosis & hemosiderin deposits; focal areas of congestion & consolidation with reactive pneumocytes
**12**	1	0			0			0		1	Localized alveolar hemorrhage with fibrin and hemosiderin‐laden macrophages
**13**	5	3	Epithelial	Viable	0			1	Foci of foreign body type giant cell reactions; peribronchiolar lymphoid aggregates	1	Focal areas of hemorrhage; single sub‐mm foreign body with refractility
**14**	4	0			1	Predominant Fetal	Viable	1	Non‐occlusive arterial reactive changes with thickening and remodeling	2	Focal intra‐arterial fibrous occlusion; focal small capillary vascular proliferation; small subpleural/septal LN and minor reactive vascular changes; focal osseous metaplasia
**15**	12	11	Embryonal, undifferentiated, fetal, mesenchymal	40–100	1	Embryonal, undifferentiated	100	0		0	
**16**	1	1	Fetal, embryonal, macrotrabecular, small cell		0			0		0	
**17**	2	0			0			0		2	Multinucleated giant cells; necrotizing granuloma
**18**	37	34	Fetal, embryonal	35–90	0			3	Crush and processing artifact	0	
**19**	3	0			0			3	Normal lung parenchyma; focal arterial intimal to intramural dystrophic calcifications	0	
**20**	1	1	Fetal, embryonal, macrotrabecular, pleomorphic	100	0			0		0	
**21**	3	2	Fetal, embryonal, macrotrabecular	20–90	1	Fetal, embryonal	5	0		0	

Abbreviation: LN, lymph node.

Specimens resected represents all specimens removed across all surgeries performed during the study period. All present subtypes of HB are listed, as well as all available viability scoring. Diagnoses are included for the HB‐negative specimens that were resected.

#### 3.1.3 Hospitalization and long‐term outcomes

Post‐operative and follow‐up data are summarized in Table 1. Of seven patients requiring ICU admission, six underwent thoracotomy with mean length of stay (LOS) of 1.57 days. Mean duration of ChT drainage was 1.52 days. Post‐operative complications included one episode of tracheitis in a patient with a chronic tracheostomy and one episode of pneumothorax requiring ChT replacement.

Four patients are deceased due to relapse. Four are alive with disease (AWD) and 13 are alive with no evidence of disease (NED). Additionally, long‐term outcomes were reviewed for 13 patients who had fewer specimens resected than number visualized on preoperative imaging. Nine have NED, all of whom underwent thoracoscopy. The remaining four patients had miliary disease and underwent thoracotomy. Two are deceased from disease progression and two are AWD. Of note, one additional patient with miliary disease is alive with NED.

### 3.2 Thoracotomy versus thoracoscopy

The metastatic cohort was separated by operative approach (thoracoscopy, n_operation_ = 12 and thoracotomy n_operation_ = 17) to evaluate potential differences in outcome. Two patients underwent thoracotomy for their first operation but then underwent thoracoscopy for subsequent operations; therefore, these patients were excluded from survival analysis.

#### 3.2.1 Pathologic evaluation

Twelve thoracoscopic operations yielded 28 specimens, 19 containing HB. Fourteen ICG‐avid specimens were pathologically positive, yielding a sensitivity of 73.7%. Four lesions were ICG‐avid but pathologically negative, yielding a specificity of 55.6%. The PPV and NPV were 77.8% and 50%, respectively. The mean number of lesions resected in the thoracoscopic group was two. (Table 3).

Ninety‐two specimens were resected via 17 thoracotomies with 66 containing HB. Sixty‐three of these specimens were ICG‐avid, yielding a sensitivity of 95.4%. Specificity was 57.7%. The resultant PPV and NPV were 85.1% and 83%, respectively. The mean number of lesions resected was 3.7. The number of specimens removed in each operative approach groups did not differ (p‐value=.09). The diagnostic accuracy of ICG between thoracoscopy and thoracotomy was not statistically significant (*p*‐value = 0.43). (Table 3).

#### 3.2.2 Hospitalization and long‐term outcomes

Patient age at time of surgery, days on the SS, and ChT duration was equivalent. However, HD duration was longer in the thoracotomy group (10.4 vs 5.5, *p*‐value = 0.02). OS was 66% in the thoracotomy cohort with 75% of patients AWD. In the thoracoscopy cohort, OS and patient AWD were 100% and 71%, respectively. (Table 1).

### 3.3 Primary versus relapsed disease

#### 3.3.1 Pathologic evaluation

Primary disease is defined as a patient on therapy since diagnosis and never in remission, including progressive disease. Relapsed disease is defined as a patient previously in remission, off therapy, when s/he develops a new focus of disease.

In 11 patients with primary disease, 201 nodules were identified on preoperative imaging (mean 5.5 lesions per patient, range of 0–59). Eighteen operations were performed, and 99 specimens removed. Seventy were HB‐positive, 66 of which were ICG‐avid, yielding a sensitivity of 94.3%. Twelve ICG‐avid specimens were pathologically negative, yielding a specificity of 58.6%. PPV and NPV were 84.6% and 81%, respectively. A mean of 4.1 lesions were removed per operation. (Table 3).

In 10 patients with relapsed disease, 24 nodules were identified on preoperative imaging (mean 2.18 lesions per patient, range of 0–4). Eleven operations were performed, and 21 specimens removed. Fifteen were HB‐positive, 11 of which were ICG‐avid, yielding a sensitivity of 73.3%. Specificity was 50%. PPV and NPV were 78.6% and 42.9%, respectively. Fewer specimens were removed per operation from patients with relapse (mean 1.7 lesions, *p*‐value = 0.04). The diagnostic accuracy of ICG was not significantly different between patients with primary and relapsed disease (*p*‐value = 0.71). (Table 3).

#### 3.3.2 Long‐term outcomes

The OS for the primary cohort was 72.7% with a mean follow‐up of 174.5 days; 75% are AWD. The OS for the relapsed cohort was 90% with a mean follow‐up of 406.9 days; 78% are AWD. OS for patients with primary disease was not different from those with relapsed disease (*p*‐value = 0.17). (Table 1).

### 3.4 PDE‐Neo versus Stryker

Due to availability, two separate camera imaging systems were used during this study period. Initially, the PDE‐Neo was the only available fluorescent capable imaging device and was used for the first eight surgeries on seven patients. The PDE‐Neo does not have minimally invasive capabilities and is only compatible with an open procedure. Twenty‐seven specimens were removed using the PDE‐Neo system. Of the 12 specimens that contained HB, 11 were ICG avid translating to a sensitivity of 91.7% and a specificity of 80%. The PPV and NPV were 78.6 and 92.3%, respectively. (Table 3).

The remaining 21 procedures in this review were completed using the Stryker fluorescent capable camera system. Overall, 93 specimens were resected, of which 73 were HB‐positive and 78 specimens demonstrated ICG‐avidity. The overall sensitivity of 90.4% and specificity of 40%; the PPV and NPV were 84.6 and 53.3%, respectively. The difference between the two camera systems was not statistically significant (*p* = 0.44). (Table 3).

We further stratified the Stryker group into the specimens resected via thoracotomy using the SPY‐PHI technology and thoracoscopic removal using the 1588‐AIM. Sixty‐five specimens were resected in the Stryker thoracotomy group; 54 specimens were HB‐positive and 60 specimens were ICG‐avid. Sensitivity and specificity with the SPY‐PHI were 96.3 and 27.3%, respectively. PPV and NPV were up to 86.7 and 60%, respectively. Twenty‐eight specimens were removed using the Stryker 1588‐AIM for thoracoscopy; 19 were HB‐positive while 18 specimens were ICG‐avid yielding a sensitivity and specificity of 73.7 and 55.6%, respectively. PPV and NPV were 77.8 and 50%, respectively. The difference between the PDE‐Neo, SPY‐PHI and 1588‐AIM camera types was not statistically significant (*p* = 0.75). (Table 3).

### 3.5 Intra‐abdominal disease

Nine patients underwent 11 hepatic resections (Table 5). Two patients underwent three operations for relapsed disease while eight had primary disease; all were performed via laparotomy. Three procedures consisted of wedge resections alone, while four combined wedge resections with a formal hepatectomy. Over a third of the operations were extended resections. Twenty‐five specimens were removed (mean 2.27 per operation, range of 1–6, Table 5).

**TABLE 5:** Patient and disease characteristics, monitoring, treatment type, and outcomes for abdominal operations

Patient	Age (years)/ Gender	Primary (P) v. Relapse (R)	PRE‐TEXT	Preoperative Chemotherapy (# of cycles)	POST‐TEXT at Surgery	ICG days prior to OR	Type of Surgery	# of Specimens Resected/ ICG+/HB+	Margin	Number of Hospital Days			Complication	AFP at Surgery/30 Day Postop	Follow‐up Days	Outcome
										ICU	Surg	Onc				
1	5.21/F	R	N/A	Cisplatin/doxorubicin x2 Ifosfamide/carboplatin/etoposide x3 Irinotecan x9	N/A^a^	3	Wedge resection of segments 5/6 and the cut surface of the liver	2/2/1	Negative	10				4742.5/4270	139	Deceased
										0	10	0				
2	3.4/M	P	IV	Cisplatin/vincristine/doxorubicin/5‐FU x6 Vincristine/irinotecan x7	III	1	Right trisectionectomy, wedge resection segment 3, biopsy of segments 2–3	3/3/1	Negative	9				24795.3/830.3	332	Alive with disease
										1	7	0				
3	2.19/M	P	II	Cisplatin/doxorubicin x3 Vincristine/irinotecan x1	II	4	Right hemihepatectomy	1/1/1	Negative	6			Transfusion	920.1/153.2	223	Alive with disease
										2	3	0				
4a	8.22/F	P	II	Cisplatin x2	II	3	Segment 3 biopsy, temporary abdominal closure	2/0/0	Negative	51			Temporary abdominal closure, hyperkalemia	25082/38.2	214	Alive without disease
										8	5	5				
4b						4	Left hemihepatectomy	1/0/1		same admission as 4a				same as above		
		
5	1.12/F	P	II	Cisplatin/doxorubicin x3 Carboplatin/doxorubicin x2	II	3	Right hemihepatectomy	1/1/1	Positive ^e^	6			Bile duct injury, not clinically significant	28.6/25	44	Alive without disease
										1	5	0				
6a	3.59/M	R	N/A	Cisplatin/vincristine/doxorubicin/5‐FU x6 Carboplatin/etoposide x1 Cisplatin/SAHA x3	N/A^b^	3	Left lateral sectionectomy and wedge biopsies, RFA segment 8 ^d^	3/3/2	Negative	8				956.6/368.8	136	Alive with disease
										1	6	0				
6b	3.82/M			Cisplatin/SAHA x1	N/A^c^	5	Wedge resections of the right liver lobe	2/2/0	N/A	10			Culture negative pneumonia, 7 days of antibiotics	615.9/11282.8		
										0	9	0				
7	3.09/M	P	IV	Cisplatin/doxorubicin x3 Carboplatin/doxorubicin x1	III	4	Right extended hemihepatectomy wedge resections of left lateral segment, microwave ablation of left lateral section^d^	3/3/3	Focal Positive	8				13372.5/47.4	104	Alive without disease
										1	6	0				
8	.2.64/F	P	II	Cisplatin/doxorubicin x2 Cisplatin x2	II	3	Right extended hemihpatectomy	1/1/1	Negative	6				215466.8/1080	39	Alive without disease
										1	4	0				
9	11.89/M	P	III	Cisplatin x2 Cisplatin/doxorubicin/sorafenib x4 Gemcitabine/oxaliplatin/sorafenib x3	III	6	Right extended hemihepatectomy, wedge resection x5 in left lateral section	6/6/2	Negative	9				191933.4/ ^f^	8	Alive with disease
										2	6	0				

For primary liver tumors, PRETEXT (**PRE‐T**reatment **EXT**ent of disease) and POSTTEXT (**POST‐T**reatment **EXT**ent) staging was assessed. In relapsed intra‐abdominal disease, the lesion location, size, and involved liver segments were determined. The preoperative chemotherapy regimens indicate the total number of cycles received of each regimen, but are not in chronological order. ICG days prior to OR refers to the number of intervening days between ICG administration and the corresponding operation. Serum alpha‐fetoprotein (AFP) levels (ng/mL), preoperative and 30 days postoperative, are provided where available with notations made if an additional oncologic surgery was performed prior to the postoperative AFP level. Margins refers to the malignancy status of the microscopic parenchymal margin. Number of hospital days indicates the total number of days the patient was admitted for the hospitalization where the surgery occurred. Days on the ICU, surgery and oncology services indicate post‐operative days on these services, exclusively. Under complications, transfusion indicates that the patient received a blood transfusion within 72 hours following their operation. Follow‐up duration is calculated from the day of surgery or the day of first surgery for patients who underwent multiple procedures.

a, b, c: indicate relapsed disease where POST‐TEXT is not applicable; the involved segments and dimensions of tumor are provided instead.

^a^ 3.1 cm lesion in segment VI, 1.3 cm lesion on cut edge of liver.

^b^ 1.5 cm lesion in segment II, 0.7 cm lesion in segment 6, 1 cm lesion in segment 8.

^c^ No lesions on imaging.

^d^ indicates concomitant procedure with interventional radiology.

^e^ denotes tumor that extended to the microscopic margin.

^f^ 30 day AFP was not available.

#### 3.5.1 Pathologic evaluation

**FIGURE 5:**
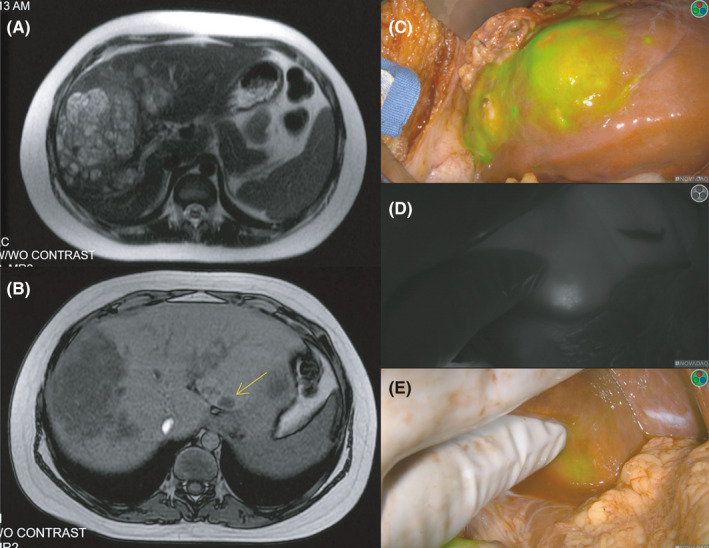
CT and intraoperative imaging of ICG‐positive abdominal lesions. The left panel (A‐B) demonstrates the preoperative CT in axial views. Panel a shows the T2‐weighted image of the right sided lesions; Panel b shows the multifocality of this tumor indicated by the yellow arrow in the T1‐weighted image. The right (C‐E) panel shows the intraoperative imaging during laparotomy using the Pinpoint ICG system. Panel c demonstrates the ICG overlay of the large right‐sided lesion. Panel d and e exhibit the ICG‐avidity of a smaller left‐sided lesion with the black and white (D) and ICG overlay (E)

Thirteen of 25 resected specimens were HB positive, and four contained multiple lesions. Twelve of 13 were ICG‐avid, yielding a sensitivity of 92.3% (Figure 5). Ten lesions were ICG‐avid but negative for malignancy, yielding a specificity of 16.7% (Table 3). The OR for an ICG‐avid lesion to be pathologically positive was 2.42 (CI 0.10–58.18; *p*‐value = 0.56). The most common histologic findings for FP were inflammation and steatosis. Of 13 HB‐positive specimens, all parenchymal margins were grossly negative. Two lesions had microscopically positive margins described as focally positive or as small nests of cells extending to the margin (Table 5).

In six cases multiple ICG‐avid specimens were removed, two of which were for relapsed disease with multiple wedge resections. Of the 15 ICG‐avid wedge resections, five were HB‐positive. Two of the HB‐positive lesions were not detected by preoperative imaging or intra‐operative palpation/visualization, but were resected solely on ICG‐avidity. One HB‐positive lesion was ICG‐avid, noticeable on preoperative imaging and visualized on intraoperative imaging. The final two HB‐positive lesions were ICG‐avid but were also palpable during examination of the liver even though they were not detected on preoperative imaging. The remaining ten ICG‐avid, HB‐negative wedge resections were not palpable; however, six were detected by ICG‐avidity alone and four were visualized on preoperative imaging. Intraoperative ultrasound (IOUS) was not routinely used to identify lesions for the patients included in this retrospective review.

Finally, three patients possessed a POSTTEXT III tumor but also underwent wedge resections in the liver remnant in addition to the extended hepatectomy. Both wedge resections for patient #7 were palpable and ICG‐avid. In patients #2 and #9, the lesions were not palpable or visible but ICG‐avid. IOUS was only utilized for patient #9 and did not demonstrate correlate lesions to areas of ICG‐avidity. Although none of these lesions were seen on preoperative imaging, three of nine were HB‐positive.

#### 3.5.2 Intra‐operative complications, hospitalization data, long‐term outcomes

Intra‐operative data, post‐operative course and long‐term outcomes are summarized in Table 5. Notably, one patient's blood transfusion requirements resulted in hemodynamic instability and hyperkalemia and necessitated temporary abdominal closure and return to the OR. Another patient incurred a small common bile duct injury that was recognized and repaired without residual leak on completion cholangiogram. The median number of HD was 8.5 (range, 6–51). There were no major complications following resection. Overall, eight of nine patients are alive with a mean follow‐up of 138 days. Four are AWD. The patient who died experienced progressive relapsed disease.

## 4 DISCUSSION

ICG has been identified as an adjunct for complete resection of both primary and metastatic hepatoblastoma. Case series have described ICG use in primary hepatic tumor resections, but the effect on resection margin, as well as OS and EFS have yet to be delineated.^16^, ^17^, ^18^ ICG for HB pulmonary metastasectomy has been described including 22 patients who underwent 62 procedures.^15^, ^16^, ^17^, ^19^, ^20^ In this study, we present the largest cohort of ICG use in both primary and metastatic HB.

Sensitivity of ICG in our thoracic patient population was 91% with a specificity of 57%. Almost half of the cases had an HB‐positive lesion resected based on ICG‐avidity alone. Three patients had disease that was not seen on pre‐operative imaging or intra‐operatively. Without ICG localization, these lesions may have gone unnoticed. Given its favorable side‐effect profile, ICG is a beneficial adjunct in metastatic disease.

There were eight false negatives (FN) occurrences in seven surgeries, three in thoracotomies and five in thoracoscopies. In five of these operations there were concomitant resected lesions with ICG uptake while the remaining two operations did not demonstrate ICG‐avidity in any of the removed specimens. This may represent unsuccessful injection or tumor characteristics that are not amenable to ICG uptake. However, while potential FN may occur, the high sensitivity of ICG demonstrates FN are the exception instead of the rule.

FP occurred in areas containing hemorrhage, inflammation or blood vessel irregularity (Table 4). This may reflect ICG’s binding of serum proteins with escape from capillaries. Interestingly, ICG demonstrated increased sensitivity and NPV in primary disease and thoracotomy when compared to relapsed disease and thoracoscopy, respectively.

The increased sensitivity of ICG in the thoracotomy cohort may be influenced by several factors. Of note, while not statistically significant, the thoracotomy cohort had more specimens which suggest a greater burden of disease and may impart bias due to increased power in this cohort. Additionally, ICG penetrance is a known limitation of its use. ICG‐avidity is difficult to detect if greater than 1.0 cm from the parenchymal surface.^15^, ^21^ For example, two specimens from separate patients were ICG‐negative in‐situ but demonstrated ICG‐avidity ex‐vivo; one was noted to be 7 mm from the pleural surface on imaging. While HB lesions are not commonly palpable, open surgery provides an enhanced ability to manipulate tissue and expose ICG‐avidity within the lung parenchyma, likely contributing to the increased sensitivity (Figure 1). If the lesions are palpable, thoracotomy provides increased tactile feedback compared to thoracoscopy. Due to these factors, success of thoracoscopy relies on optimization of the operative conditions, specifically adequate lung isolation and deflation all of which could affect the ability to detect ICG‐avidity. Another potential confounding factor is that nine operations were initiated thoracoscopically but converted to thoracotomy, often to improve visualization or palpation based on the operating surgeon's judgement. This may represent a selection bias between techniques, as these surgeries were placed exclusively in the thoracotomy category and do not account for ICG‐avidity seen thoracoscopically. Finally, an additional notable confounding factor was initial equipment limitation only allowing for ICG use during thoracotomy contributing to the larger size of this cohort. Outside of the PDE‐Neo group, only one patient fell into the strict thoracotomy category; the remainder were conversions from an initial thoracoscopic procedure. All of these factors may contribute to the difference in diagnostic accuracy displayed in this study. As either surgical approach produced an inordinate number of post‐operative complications or difference in survival, the authors feel thoracoscopy utilizing ICG is viable method for HB resection in certain patient populations, specifically in patients with decreased burden of disease on preoperative imaging who may be spared the morbidity associated with thoracotomy. The choice of surgical procedure should always be based on the individual patient and surgeon's confidence to obtain a complete resection.

Two separate operating systems were utilized during this study. The PDE‐Neo system demonstrates an increased specificity and NPV compared to the Stryker system; however, this difference was not statistically significant likely because it was underpowered. The authors have elected to use the Stryker system because of the functionality of the equipment. In our experience, the PDE‐Neo device is bulkier making it more difficult to manipulate especially in the smaller surgical fields. The Stryker system also provides the green overlay mode allowing for real time visualization of the surgical field and existing fluorescence; the PDE‐Neo did not provide overlay and fluorescence could only be visualized in black and white mode. In the authors’ opinion, these features increased the intraoperative ability to identify areas of ICG‐avidity compared to the PDE‐Neo system. Ultimately, the enhanced visualization of ICG‐avidity may result in a greater number of FPs. However, there were not more than three FP specimens removed from a single patient and no complications related to additional resections. Therefore, the benefits of the Stryker system outweighed the potential of a FP resection. Finally, Stryker allows for a thoracoscopic fluorescence evaluation sparing the patient the necessity of a thoracotomy to use fluorescent imaging. For these reasons, the authors have elected to use Stryker fluorescence capable devices.

Increased ICG sensitivity was also noted in the primary disease group compared to patients with relapse, and more lesions were resected from these patients. Again, fewer specimens excised from the relapse group may affect the power to determine an accurate sensitivity, specificity, PPV and NPV. Additionally, a physiologic difference in tumor cells of relapsed patients may affect their ability to retain ICG, either in uptake or excretion. However, this observation requires further study to elucidate potential molecular differences. The difference in sensitivity between the two groups should not preclude ICG use in the relapsed population. The majority of ICG‐avid relapsed specimens revealed tumor cells on pathology with only three FPs described. Again, there was no difference in survival and NED between groups.

This cohort also highlights the limitation of imaging for lesion localization. On average, imaging was completed within two weeks of surgery; however, over half of the operations did not localize all lesions noted on imaging (Table 2). One explanation involves the ongoing chemotherapeutic effect on metastases as highlighted in one patient where preoperative imaging demonstrated 40 lesions in the right lower lobe, however, pathologic examination of the lobar specimen identified 28 HB foci. This also demonstrates the unique challenge that miliary disease poses. Effective chemotherapy with judicious resection remains of utmost importance; however, it is difficult to rely solely on cross sectional imaging. Eighty percent of the patients with miliary disease had HB identified by ICG‐avidity, and in half of these patients a focus HB was indicated by ICG‐avidity alone intraoperatively. This emphasizes the importance of developing reliable adjuncts, such as ICG, to facilitate disease identification and resection by a multi‐disciplinary pediatric oncology team. In the authors’ opinion, ICG provides increased confidence in a complete oncologic resection for metastatic disease when used as an adjunct to the preoperative imaging and intraoperative assessment.

In the abdominal cohort, ICG proved sensitive for detecting HB but lacked specificity. The decreased specificity was due to FP in areas of inflammation or steatosis. Generally, HB patients are less likely to have fatty liver disease since prevalence increases with age, however, this finding may be present in older patients.^22^ The concern for FP is most pronounced when ICG‐avidity is distant from the dominant lesion because it may require a more extensive resection or transplant. In this study, distant ICG‐avid lesions did not change the type of operation performed and were removed by wedge resection. There were distant lesions positive for HB, indicating multifocal disease; however, the majority were pathologically negative for malignancy. The multifocal lesions removed in this study were superficial and resected minimal hepatic tissue from the liver remnant. If there were concerns for deeper lesions, these areas were not resected but instead were radioablated by interventional radiology as indicated (Figure 5). The decision to remove multifocal lesions in the liver remnant is based on extensive knowledge of the patient by the attending surgeon and intraoperative judgement. When necessary, multidisciplinary intraoperative consults were pursued which included pediatric oncology and interventional radiology highlighting the importance of access to this specialized team. The MR imaging is an additional helpful adjunct; however, in the setting of multifocal disease the sensitivity is not proven. The majority of wedge resections demonstrated supplementary correlates to indicate removal (palpation, intraoperative visualization, preoperative imaging). However, of the remaining lesions removed solely for ICG avidity, 25% contained disease. Importantly, there were no complications from additional wedge resections. In our opinion, as a tertiary center for complex HB referrals, superficial wedge resections to remove ICG‐avid lesions are appropriate if there is clinical concern for disease on a patient‐by‐patient basis.

Only one patient demonstrated a lack of ICG‐avidity. Patient #4 was planned for a left hemihepatectomy. During the initial stages of the operation there were concerning nodules that were biopsied. After initiating the hemihepatectomy, the patient became unstable due to hyperkalemia and the procedure was aborted with a temporary abdominal closure. After overnight stabilization the patient returned to the OR for completion of the resection. Although, ICG was given with optimal timing three days prior to resection, there was no evidence of avidity highlighting the potential of an unsuccessful injection and the cause of the one FN within this cohort.

Finally, ICG assisted in delineating surgical margins. There were no grossly positive margins, however, microscopically positive margins were detected in two patients. Patient #5 illustrates the utility of ICG in this setting. In each case the proposed liver remnant was inspected for ICG uptake prior to and following the resection. Patient #5 had a large single lesion; after mapping the projected hepatic resection margin, there was persistent ICG uptake medial to the planned remnant. In light of this finding, the margin was extended into the caudate and following resection the liver did not demonstrate any additional avidity. The pathologic assessment demonstrated positive microscopic margins. In this patient, if the additional rim of tissue was not removed these cancer cells would remain in vivo serving as a potential nidus for recurrence. The implementation of this technique continues to rely on surgeon judgement and experience of when additional resection is necessary.

In conclusion, our data indicates that ICG is a useful adjunct for achieving disease control in HB. In hepatic disease, it clarified resection margins on dominant lesions and identified areas of multifocal disease. One important caveat for hepatic resection, specifically in potential multifocal disease, remains thoughtful administration of ICG to limit non‐specific background fluorescence; although it varied in our study due to patient availability, ideal timing is three to four days prior to surgery. In the thoracic cohort, ICG aided in the detection of non‐palpable tumor deposits not visualized on pre‐operative imaging. It should not replace preoperative imaging and intraoperative inspection and palpation but serves to complement these techniques to improve the possibility of a complete resection. It offers the added benefit of identifying small lesions, the smallest in our cohort was <1 mm; however, other techniques are necessary to identify deeper lesions and those lacking ICG‐avidity to avoid FN. Although, this is the largest published cohort of ICG use in HB, sample size is small due to rarity of disease. Additional patients and specimens will allow us to elucidate if there is truly a difference in ICG sensitivity between surgical approaches and type of patient disease. Nonetheless, we had no adverse outcomes associated with ICG use and report an OS of 81% at a mean of 9.5 months in our patients with metastatic disease and 89% survival at a mean of 4.5 months after hepatic resection.

## CONFLICTS OF INTEREST

None declared.

## AUTHOR CONTRIBUTIONS

Charissa M. Lake: data curation, investigation, methodology, formal analysis, visualization, writing—original draft, writing—review and editing. Alexander J. Bondoc: conceptualization, data curation, investigation, methodology, project administration, supervision, resources, writing—review and editing. A. Roshni Dasgupta: conceptualization, supervision, writing—review and editing. Todd M. Jenkins: formal analysis, resources, validation, writing—review and editing. Alexander J. Towbin: investigation, resources, writing—review and editing. Ethan A. Smith: investigation, resources, writing—review and editing. Maria H. Alonso: conceptualization, resources, writing—review and editing. James I. Geller: conceptualization, resources, writing—review and editing. Gregory M. Tiao: conceptualization, methodology, project administration, resources, supervision, writing—review and editing.

## Data Availability

Data available on request from the authors. The data that support the findings of this study are available from the corresponding author upon reasonable request.
